# Chlorinated biphenyls: MAK Value Documentation, addendum – Translation of the German version from 2025

**DOI:** 10.34865/mb0cbphpcbe11_1ad

**Published:** 2026-03-30

**Authors:** Andrea Hartwig, MAK Commission

**Affiliations:** 1 Institute of Applied Biosciences, Department of Food Chemistry and Toxicology, Karlsruhe Institute of Technology (KIT) Adenauerring 20a, Building 50.41 76131 Karlsruhe Germany; 2 Permanent Senate Commission for the Investigation of Health Hazards of Chemical Compounds in the Work Area, Deutsche Forschungsgemeinschaft, Kennedyallee 40, 53175 Bonn, Germany. Further information: Permanent Senate Commission for the Investigation of Health Hazards of Chemical Compounds in the Work Area | DFG

**Keywords:** chlorinated biphenyls, developmental toxicity, MAK value, maximum workplace concentration, pregnancy risk group, Luft, air

## Abstract

The German Senate Commission for the Investigation of Health Hazards of Chemical Compounds in the Work Area (MAK Commission) derived a concentration in workplace air for chlorinated biphenyls (PCBs) [1336-36-3] which is without risk to the developing foetus (corresponding to Pregnancy Risk Group C). The derivation is based on the prerequisite for the classification in Pregnancy Risk Group C at 3.5 μg/l plasma for the sum of the 6 indicator congeners (6IC) PCB 28, PCB 52, PCB 101, PCB 138, PCB 153 and PCB 180. Assuming a background concentration of 2 μg 6IC/l plasma for women up to 45 years of age with data from about 2010, an increment of 1.5 μg 6IC/l plasma is thus tolerable. This increment was estimated to correspond to a total PCB concentration in air of 0.0008 mg/m^3^ measured as the sum of the 6IC multiplied by 5. This concentration represents a rather conservative estimate, as the background concentration in plasma has reduced since and a respiratory volume of 10 m^3^ has been assumed, which might overpredict the exposure at workplaces in PCB-contaminated office or public buildings. Additionally, age and duration of exposure are important determinants for the inner exposure. These factors should be considered when evaluating the measurement results.

**Table:** 

**MAK value (2015)**	**0.003 mg/m^3^ I^a)^ (inhalable fraction)**
**Peak limitation (2015)**	**Category II, excursion factor 8**
	
**Absorption through the skin (2012)**	**H**
**Sensitization**	**–**
**Carcinogenicity (2015)**	**Category 4**
**Prenatal toxicity (2015)**	**Pregnancy Risk Group B^b)^**
**Germ cell mutagenicity (2015)**	**Category 5**
	
**BAT value (2015)**	**15 µg/l plasma for Σ PCB 28, PCB 52, PCB 101, PCB 138, PCB 153, PCB 180^c)^**
**BAR (2011)**	**PCB 28: 0.02 μg/l plasma** **PCB 52: < 0.01 μg/l plasma** **PCB 101: < 0.01 μg/l plasma**
CAS number	1336-36-3

^a)^ (PCB 28 + PCB 52 + PCB 101 + PCB 138 + PCB 153 + PCB 180) × 5 corresponds to total PCBs in air

^b)^ prerequisite for assignment to Pregnancy Risk Group C (see “Manifesto”)

^c)^ corresponds to total PCBs in plasma

## Prerequisite for assignment to Pregnancy Risk Group C

This addendum derives the concentration in workplace air at which PCBs would be assigned to Pregnancy Risk Group C.

Based on extensive epidemiological studies of the most sensitive end points developmental neurotoxicity and reduced birth weights, as well as animal data, a plasma concentration at which no damage to the foetus is to be expected of 3.5 µg PCB indicator congeners/l has been derived. The concentration of 3.5 µg/l plasma for the sum of the 6 indicator congeners (6IC) (PCB 28, PCB 52, PCB 101, PCB 138, PCB 153 and PCB 180) is therefore considered to be the prerequisite for assignment to Pregnancy Risk Group C (Brinkmann et al. 2020).

The MAK value of **0.003 mg total PCBs/m^3^** was determined based on the effects of dioxin-like PCBs. In order to assess also the effects of non-dioxin-like PCBs, a concentration was calculated for the 6IC in air based on the NOAEL (no observed adverse effect level) for PCB 153 of 17 µg/l plasma. The background concentration of 8 µg total PCBs/l blood (= 8 µg/l plasma for the 6IC; maximum background concentration in 1999) was taken into account. An additional plasma concentration of 9 µg IC/l of non-dioxin-like PCBs is not likely to be reached if the MAK value is observed. With a half-life of 12 years for the **higher chlorinated **PCBs (PCB 138, PCB 153 and PCB 180), it was calculated that 9 µg of **higher chlorinated** IC/l plasma corresponds to a concentration of 0.833 µg of **higher chlorinated** IC/m^3^ (respiratory volume of 10 m^3^ per day, exposure on 5 days/week, 100% absorption by inhalation, half-life of 12 years). However, since air contains mainly low-chlorinated PCBs with a shorter half-life, which do not accumulate to the same extent, the MAK value protects also against the effects of non-dioxin-like PCBs (Hartwig and MAK Commission 2016).

The background value of 8 µg/l plasma for the sum of the 6IC is very conservative, as background exposure has decreased significantly since 1999. In an evaluation of data from around 2010, the 95^th^ percentile of the background concentration for 36 to 45-year-old men and women was 1.95 µg/l plasma for the sum of PCB 138, PCB 153 and PCB 180 (Table 1). No significant sex difference was found (Schettgen et al. 2015).

**Tab. 1:** Plasma concentrations [µg/l] of PCBs in the general adult population in Germany (Schettgen et al. 2015)

**PCB congener**	**Value measured**	**Age [years]**				
		**18–25**	**26–35**	**36–45**	**46–55**	**56–65**
		n = 157	n = 710	n = 400	n = 525	n = 357
PCB 138	median	0.12	0.15	0.24	0.39	0.56
	95^th^ percentile	0.25	0.33	0.53	0.93	1.26
	maximum value	3.1	0.71	1.08	1.70	3.98
PCB 153	median	0.17	0.21	0.37	0.63	0.92
	95^th^ percentile	0.38	0.49	0.79	1.41	1.94
	maximum value	0.84	0.89	1.55	2.65	5.45
PCB 180	median	0.10	0.14	0.29	0.57	0.87
	95^th^ percentile	0.29	0.34	0.65	1.23	1.87
	maximum value	0.40	0.77	1.43	4.59	9.08
PCB 138 + PCB 153 + PCB 180	median	0.38	0.50	0.92	1.58	2.41
	95^th^ percentile	0.88	1.14	**1.95**	3.54	4.82
	maximum value	1.80	2.37	3.57	8.19	18.5

The sum of the concentrations of PCB 138, PCB 153 and PCB 180 corresponds approximately to the concentration of the 6IC in the plasma of persons not exposed to PCBs by inhalation (Table 2 and 3), as the other 3 IC are virtually not detectable in these persons.

However, the internal exposure of people over the age of 45 is still high compared with that of younger adults. This age group is not relevant for the assessment of developmental toxicity, however, as very few mothers give birth over the age of 45 (0.2% of the total number of births; in 2023: 1810 births to mothers over 45 years of age (Statistisches Bundesamt 2024 b) of a total of 692 989 births in Germany (Statistisches Bundesamt 2024 a)). Therefore, the background concentration of the 36 to 45 age group of about 2 µg 6IC/l plasma is used as the basis for assessing the developmental toxicity. The permitted increment, in order not to exceed a level of 3.5 µg 6IC/l plasma, is therefore 1.5 µg 6IC/l plasma (corresponding to 3 µg total PCBs/l plasma = 1.5 µg total PCBs/l blood).

During inhalation exposure, mainly volatile PCBs are expected to be present in the air (about 95% at the 95^th^ percentiles in Table 2).

**Tab. 2:** Concentrations of the 6IC in individuals exposed via the air and in control persons (Schettgen et al. 2012)

**Value measured**	**PCB congener**						
	**28**	**52**	**101**	**138**	**153**	**180**	**6IC × 5**
	concentration in the air [ng/m^3^]						
median	140	160	29	3	2	< 1	1740
95^th^ percentile	320	348	86	22	13	2	3740
maximum value	450	470	150	31	21	3	4280
	concentration in the blood of exposed persons [µg/l plasma]						
median	0.087	0.024	0.012	0.253	0.380	0.279	
95^th^ percentile	0.352	0.091	0.046	0.846	1.256	1.085	
maximum value	0.878	0.426	0.124	2.226	3.360	3.179	
	concentration in the blood of the control persons [µg/l plasma]						
median	< 0.01	< 0.01	< 0.01	0.263	0.392	0.301	
95^th^ percentile	0.021	< 0.01	< 0.01	0.92	1.492	1.148	
maximum value	0.059	0.029	0.015	2.437	3.523	3.186	

The half-lives of the more volatile PCB 28, PCB 52 and PCB 101 are significantly shorter (2.4, 1.0 and 1.3 years, respectively) than those of the highly chlorinated, less volatile PCBs (12 years) (Esser et al. 2021). Assuming an average half-life of 2 years for the more volatile PCBs, the external concentration required to achieve a steady state (one-compartment model) of 1.5 µg total PCBs/l blood (= 1.5 µg 6IC/l plasma) can be calculated as follows:

1.5 µg total PCBs/l blood × 300 (fat : blood partition coefficient) = 450 µg total PCBs/kg fat; assuming 20% body fat, this results in 90 µg total PCBs/kg body weight. With a half-life of 2 years (730 days), this concentration is reached with a daily intake of 85 ng/kg body weight and day (ln2 × 90 µg/kg body weight/730 days). Under workplace conditions (10 m^3^, 100% absorption by inhalation, 70 kg body weight, 5 days per week), this corresponds to a concentration of 0.833 µg total PCBs/m^3^ (85 ng/kg body weight × 7/5 × 70 kg/10 m^3^).

As already described above, it is assumed that the additional exposure is caused only by volatile PCBs, which has been largely confirmed by measurements (Table 2 and 3).

The study of Meyer et al. (2013) includes also data for individuals exposed by inhalation (median exposure duration 12 years in PCB-contaminated homes from the 1970s; exposure time during the week 16 hours per day; median exposure in 2011: 0.000859 mg total PCBs/m^3^) and individuals not exposed (PCB concentration < detection limit). According to the above calculation, the concentration of 0.000859 mg total PCBs/m^3^ would lead to an increment of about 1.55 µg 6IC/l plasma (1.5 × 0.859/0.833). Those exposed by inhalation had a median increment of 1.91 µg 6IC/l plasma. The calculation assumes that the respiratory volume of the residents in 1 week is the same as that during 5 days of workplace exposure (10 m^3^/day). However, the exposure of residents per week is likely to be higher, as domestic exposure on weekends can also be assumed. It is therefore assumed that they are present 16 hours per day, 7 days per week, with a resting respiratory volume of 10 m^3^ in 16 hours. This gives a predicted increment in PCB exposure of 7/5 × 1.55 µg 6IC/l plasma = 2.17 µg 6IC/l plasma.

**Tab. 3:** Concentrations of the 6IC in individuals exposed via the air and in control persons (Meyer et al. 2013)

**Value measured**	**PCB congener**						
	**28**	**52**	**101**	**138**	**153**	**180**	**6IC × 5**
	concentration in the air [ng/m^3^]						
median	61.4	94.6	8.9	0	0	0	859
	concentration in the blood of exposed persons [µg/l Plasma]						6IC
median	1.371	0.216	0.034	0.157	0.392	0.341	2.715
	concentration in the blood of control persons [µg/l Plasma]						6IC
median	0.014	< LOD	< LOD	0.134	0.346	0.262	0.805

LOD: limit of detection

The PCB increment calculated above therefore corresponds roughly to the values measured in the study of Meyer et al. (2013).

However, the relationship between external and internal exposure in the study of Schettgen et al. (2012) (exposure while working in a PCB-contaminated public building, 209 exposed individuals, 98 control persons) does not correspond to that in the study of Meyer et al. (2013) (Table 2). Here, the median air concentration of total PCBs was 1.74 µg/m^3^, almost twice as high as that in Meyer et al. (2013), but the increment of the medians for PCB 28 + PCB 52 + PCB 101 in plasma was only 0.123 µg/l compared with the value in the controls. The 3 higher chlorinated IC were not included in the calculation of increments because the control persons had higher values than the exposed persons.

The calculation performed above roughly corresponds to the data from the study of Meyer et al. (2013) and thus tends to overestimate the PCB concentration in blood compared with the results of the study of Schettgen et al. (2012).

## Manifesto

Based on the notified prerequisite for assignment to Pregnancy Risk Group C at 3.5 µg/l plasma for the 6IC PCB 28, PCB 52, PCB 101, PCB 138, PCB 153 and PCB 180 and a background concentration of 2 µg/l plasma for the 6IC, a risk to the developing foetus is not assumed at 0.0008 mg total PCBs/m^3^ (= 6IC × 5). However, higher concentrations do not necessarily indicate a risk to pregnancy, as internal exposure to substances with a very long half-life and age-dependent background exposure, such as PCBs, depends on the duration of exposure and also on the age at the start of exposure. With a half-life of 2 years, the steady state is reached after approximately 5 half-lives, that is 10 years. If the exposure period is shorter, the internal exposure is correspondingly lower. Also the PCB concentration in the blood is lower in people under the age of 45, as they have a lower background exposure. In addition, a respiratory volume of 10 m^3^ was assumed for increased physical activity, which does not apply to all workplaces, especially not in PCB-contaminated offices. Furthermore, the measurements were performed about 15 years ago. In the meantime, the background values are likely to have fallen further. These factors should be taken into consideration when evaluating the measured data.
